# Blasting Off Again: An Observational Study on Substance Use Content Exposure in Pokémon Twitch Streams

**DOI:** 10.1016/j.focus.2025.100413

**Published:** 2025-08-21

**Authors:** Matthew C. Nali, Meng Zhen Larsen, Tim K. Mackey

**Affiliations:** 1 San Diego Supercomputer Center, University of California, San Diego, San Diego, California; 2 Global Health Policy and Data Institute, San Diego, California; 3 S-3 Research, San Diego, California; 4 Global Health Program, Department of Anthropology, University of California, San Diego, San Diego, California

**Keywords:** Pokémon, twitch, alcohol, cannabis, tobacco, youth

## Abstract

**Introduction:**

The Pokémon franchise has an array of media (i.e., TV series, movies, merchandise, and Trading Card Game) that has attracted global youth and adults since its creation in 1996. Specifically, users have been known to discuss, promote, and purchase Pokémon Trading Card Game on the livestreaming platform Twitch. However, Pokémon Trading Card Game livestreaming content may also include potentially harmful content for youth and adolescents, such as depictions of substance use. Hence, the objective of this study was to assess whether popular Twitch Pokémon Trading Card Game content and influencers promote substance use topics and behavior.

**Methods:**

This study was conducted in 3 phases: (1) data mining on 2 Twitch data aggregation websites (Sullygnome.com and TwitchTracker.com) within the Pokémon Trading Card Game category; (2) data filtering using keywords through a snowball sampling strategy on the title of a Twitch stream; and (3) content coding using the WHO protocol on Internet Monitoring of Marketing of Unhealthy Products to Children and Adolescents for influencer and microinfluencer substance use marketing, including depictions of substance use or promotion of products on the streamer’s profile. The authors also conducted an assessment of any underage moderation for Twitch livestreams.

**Results:**

A total of 1,025 highly active Pokémon Trading Card Game streamers were recruited and analyzed. The use and promotion of substance use in the form of alcohol, cannabis, or tobacco was detected in 25.07% (*n*=257) of these Pokémon Trading Card Game Twitch streamers. Frequent substance use detected was alcohol (90.27%, *n*=232), followed by polysubstance use (6.61%, *n*=17), cannabis (*n*=6, 2.33%), and tobacco (*n*=2, 0.78%). Content coding detected 3 general themes, with behavioral being the most observed. This included behavioral content of substance use for the specific reason of casual use (46.06%, *n*=228) and for intoxication (26.26%, *n*=130). In addition, close to three quarters (72.76%, *n*=187) of streamers’ accounts did not prohibit underage access to account content or livestreams.

**Conclusions:**

In this study, the authors detected Twitch streamers violating Twitch community guidelines that may have exposed millions of youths and young adults in the Pokémon Trading Card Game category to different depictions of substance use. Although Twitch prohibits monetary gain in exchange for posting substance use content, the apparent lack of enforcement of Twitch community guidelines and the absence of age verification means that youth who enjoy Pokémon Trading Card Game content may also be exposed to content actively promoting substance use behaviors.

## Highlights

• Less than 25% of top Pokémon Trading Card Game (TCG) Twitch streamers promote substance use, mainly alcohol.
• A total of 73% of Pokémon TCG lack age restrictions, exposing youth to intoxication content.
• There are consistent violations of Twitch policy and weak enforcement in youth-centric TCG.

## INTRODUCTION

In 1996, Game Freak and Nintendo released Pokémon Red and Green, the first installments of what would become the Pokemon franchise. These role-playing games, in which players capture, train, and battle creatures known as Pokemon, later inspired the Japanese manga Pocket Monsters (ポケットモンスター).^1^, ^2^, ^3^, ^4^ The franchise rapidly became a global phenomenon, expanding into the animated TV series, movies, video games, and merchandise.^5^ Notably, one form of media that brought the worldwide popularity of Pokémon was the Pokémon Trading Card Game (TCG), which allowed fans to collect and battle with cards featuring various Pokémon,^6^ each with distinct attributes and abilities, encouraging strategic gameplay and engagement.

Spanning generations, children and adults compete in Pokémon TCG tournaments, divided into 3 age divisions (e.g., Junior, Senior, and Masters Division). Beyond gameplay, the cards’ real-world collectable monetary value appeals to youth and young adults, with the most expensive Pokémon card bought in 2022 by a YouTube influencer Logan Paul for $5.275 million.^6^^,^^7^ Reflecting the franchise’s massive scale, between February 2023 and March 2024, the Pokémon corporation reported producing over 64.8 billion trading cards across 93 countries and regions.^8^

In 2021, during the coronavirus disease 2019 (COVID-19) pandemic, the Pokémon TCG market surged in popularity, with card values rising by hundreds to thousands of dollars overnight.^9^, ^10^, ^11^ This increase in demand led big box retailers (e.g., Walmart and Target) to limit their TCG inventory.^12^ Owing to the decreased availability of Pokémon cards in traditional retail settings, thousands of consumers turned to secondary markets, buying cards through social streaming platforms such as Twitch, which shifted how collectibles were accessed and sold.^12^^,^^13^

Twitch, a popular livestreaming platform (owned by Amazon), has around 35 million daily users, primarily comprised of adolescents and young adults aged between 13 and 35 years.^14^ Users engage in livestreams centered on video games, music, and various creative topics, allowing users to broadcast their opinions with viewers who participate in an interactive online community.^15^, ^16^, ^17^ Twitch viewers directly interact with content creators by chatting and sending bits or donations in real time, a format distinctly different from other media, such as TV, where individuals passively consume content.^18^, ^19^, ^20^ Twitch is now a platform where millions of young users regularly actively discuss, trade, and consume content about TCG but also where other products, services, and behaviors, including those related to substance use, may be comarketed with this content.

Specifically, content related to substance use now occurs across various social media platforms (e.g., X [formerly Twitter], YouTube, TikTok, Facebook) and specifically promotes products with abuse potential (i.e., alcohol, cannabis, nicotine, tobacco, and so on) that may shape users’ perceptions, attitudes, and behaviors toward substance use risks, particularly for adolescents.^21^^,^^22^ For example, a study on TikTok found widespread promotion of cannabis use in a positive manner, with content often lacking age restrictions.^23^ Another study on Instagram found a highly interconnected network of E-cigarette brand influencers, with 75% of them not restricting their content to youth.^24^ Similarly, research on Facebook has demonstrated that users who displayed alcohol in profile images were more likely to report increased personal use.^25^ Furthermore, these displays were reinforced by peer engagement and contributed to shaping self-identity around drinking culture, where heavy consumption was framed as socially acceptable or even desirable.

For Twitch, streams may pose specific risks for youth and young adults, although only a very few studies have been conducted examining Twitch content. One study found that social media influencers on Twitch actively promoted E-cigarette use, often in exchange for compensation, with minimal platform restrictions, raising public health concerns owing to Twitch’s predominantly adolescent and young adult user base and resultant exposure to substance use content.^26^ Another study found widespread marketing of energy drinks and sugary beverage products on Twitch gaming channels, including extensive product placement and brand mentions during streams and in chat, suggesting that Twitch is a growing channel for exposure to other unhealthy products.^27^ However, no study has specifically analyzed Twitch content for influencers, advertisements, or behaviors associated with substance use topics within the Pokémon TCG Twitch community, which also has participation of and is appealing to many children and young adults.^26^^,^^27^ Hence, the objective of this study was to assess whether popular Twitch Pokémon TCG content also includes content that promotes substance use topics, products, and behavior, given the young audience for Twitch and past studies that have observed minimal content moderation on the platform.

## METHODS

This study was conducted in 3 phases: (1) data mining on 2 data aggregation websites of Twitch streamers within the Pokémon TCG category; (2) data filtering using keywords through a snowball sampling strategy on the title of a Twitch stream; and (3) content coding using the WHO protocol on Internet Monitoring of Marketing of Unhealthy Products to Children and Adolescents (WHO Protocols) for influencer and microinfluencer substance use marketing, including characterizing depictions of substance use or promotion of products on the streamer’s profile as well as assessment of any underage moderation used for Twitch livestreams.^28^^,^^29^

### Study Sample

The first phase of the study used 2 data aggregation websites for data collection to (1) identify influential and high-volume users of Twitch specific to Pokémon TCG and (2) collect historical data of archived Twitch streams for content analysis. Data aggregation sites used in this study included documentation about how they retrieve and aggregate information using the official Twitch API (application programming interface). First, the website, sullygnome (a website that performs analytical tracking across all individual streamer categories on viewership trends, channel performance, and watch and streaming time) was used to collect all available Twitch streamers who broadcast under the Pokémon TCG category.^30^ A total of 7,012 users were identified; however, only the first 1,025 users were selected because they had the highest volume of Pokémon TCG streaming hours (e.g., ≥100 hours) for the 1-year period between July 11, 2023, and July 11, 2024.^28^^,^^29^ The website TwitchTracker (an analytics platform that archives Twitch streamers’ content such as titles of streams; clips, followers; and viewership statistics, including views, peak viewership, and trends) contained aggregated statistics and content of past available streams.^31^ For each individual streamer identified on sullygnome and selected for analysis, the authors used TwitchTracker to collect data analytics on streams and then manually reviewed all stream titles that matched with their keyword list described below.

### Keyword Generation and Filtering

The second phase filtered streams for possible substance use–related content. The authors first identified 31 relevant keywords, including 3 emojis, to filter through the titles and profiles of identified Twitch streamers affiliated with the Pokémon TCG category. Keyword selection used an approach similar to a digital snowball sampling strategy (i.e., where the authors conducted manual searches and data filtering to find a keyword or a Twitch TCG creator that discussed substance use topics that subsequently led to additional keywords the authors could use for further data collection). This approach was used to identify streamers and descriptions on Twitch that made references to or used alcohol, cannabis, or tobacco while streaming (Appendix, available online, provides a full list of keywords).

### Content Analysis

The third phase was conducted through a deductive content coding approach using the WHO Protocols to identify and characterize specific themes and subthemes from selected streamers who discussed and promoted substance use topics. The deductive coding schema focused on identification of specific substance use categories and types of promotional cues. Additional emergent subthemes for the general themes characterizing why streamers included substance use content or engaged in substance use behavior were separately inductively coded owing to a lack of an existing coding framework for this type of content. First, coders manually reviewed the filtered content of influencers and microinfluencers for study inclusion criteria, which included (1) mention of substance use disorder behavior or associated product and (2) promotion of substance use in text, image, or video-related content. Two coders (first and second authors) independently reviewed the content of all 1,025 Twitch streamers for study inclusion and achieved a high intercoder reliability (κ=0.98). In case of inconsistent results, authors reviewed and conferred to reach consensus on the correct classification.

Once a Twitch user was confirmed for inclusion in the study, deductive content coding of the user’s Twitch profile and streams was annotated for (1) substance use category (i.e., type of substance depicted or used in content) and (2) promotional cues (e.g., display, brand, description, and presentation). Inductive content coding for emergent themes was used to identify and characterize (i) stated reasons or rationale for streaming substance use behavior content, (ii) specific forms of substance use behavior (e.g., intoxication, inebriation, casual use, and others), and (iii) substance use depictions and content for monetary gain generated from platform interactions (e.g., bits, subs, followers, and so on) (Table 1 code book provides details).

**Table 1:** Study Code Book on General Theme and Subtheme Definitions

General theme	General theme definition	Subtheme	Subtheme definition	Example
Behavioral	Personal use with no evident intention of monetary gain	Entertainment	Streamer mentions substance use for entertainment purposes	
		Event driven	Celebrating a holiday or personal achievement	
		Intoxication	Streamer intention to stream is exhibiting being tipsy, drunk, or hangover	
		Casual use	The streamer casually uses substances to cope with their needs	
		Content creative stream	The streamer is currently live with a guest/cohost	
Monetary gain	Substance use content that solicited monetary contributions	Bits	Virtual goods that show support for streamers that can be transferred into cash	
		Subs	Virtual goods where users can avoid in livestream advertisements for a specific user. Subscription generated can be transferred into cash	
		Followers	Interests from users to follow an individual streamer; cannot be redeemed for cash	
		Donation	Cash donated to streamer off Twitch platform	
Product promotion	Direct or indirect promotion of substance use, where the product is endorsed by a brand or exchanged for nonmonetary benefits	Direct substance marketing	The content regularly endorses or normalizes substance use	
		Indirect substance marketing	The streamer features substance use in their content but does not promote it directly	
		Personal content	Streamer promotes personal social media in exchange for substance use	
		Outside sponsor	Explicit paid sponsorship of a product by a company	

This table shows texts from Twitch stream titles showing substance use themes in Pokémon TCG streams across multiple categories.

TCG, Trading Card Game.

Finally, the authors emulated 2 user account types for assessment of age verification requirements, including (1) creating an underage Twitch account (age 14 years) and (2) accessing the platform without an account logged in. This was conducted to assess whether age restrictions applied to substance use content identified on Twitch. Specifically, these 2 methods were used to simulate whether underage users or non–logged-in users (i.e., those who do not affirm to be of the age of majority or state their age in their profile) could access streams without restriction or warning. The researchers recorded whether warnings or disclaimers would pop up on streams to deter or prevent an underage account or a non–logged-in user from accessing content.

## RESULTS

A total of 1,025 highly active streamers listed in the Twitch Pokémon TCG category were collected and analyzed. Common general observations of content generated by these streamers included discussing new card drops (i.e., product releases), live rips (i.e., booster box or individual pack openings), live battles (i.e., winner takes all cards), live giveaways (i.e., free Pokémon cards), and entertainment games (i.e., Pokémon Bingo or Pokémon gameplay) that were not formally coded or analyzed for this study.

Manual annotation and content coding per the WHO Protocol was then conducted on these highly active Pokémon TCG streamers, where 25.07% (*n*=257) were linked to content depicting the use of 1 or more substances in the form of alcohol, cannabis, tobacco, or polysubstance use of multiple substances in their Twitch TCG–related profile descriptions, associated text, or archived livestreams (Table 2). The specific types of substances discussed, depicted, used, or being advertised included alcohol (90.27%, *n*=232), cannabis (2.33%, *n*=6), tobacco (0.78%. *n*=2), and polysubstance use (6.61%, *n*=17).

**Table 2:** Summary of a Substance Use for Direct Relationship to Twitch Streamer Content

Substance use category (*n*=257)	General theme (*n*=312)	Subtheme (*n*=495)
All substance use (alcohol, cannabis, tobacco) (*n*=257, 25.07%)	Behavioral (*n*=295, 94.55%)	Entertainment (*n*=32, 6.46%)
		Event driven (*n*=63, 12.73%)
		Intoxication (*n*=130, 26.26%)
		Casual use (n=228, 46.06%)
		Content creative stream (*n*=23, 4.65%)
	Monetary gain (*n*=11, 3.53%)	Bits (*n*=1, 0.20%)
		Subs (*n*=7, 1.41%)
		Followers (*n*=1, 0.20%)
		Donation (*n*=3, 0.61%)
	Product promotion (*n*=6, 1.92%)	Direct substance marketing (*n*=1, 0.20%)
		Indirect substance marketing (*n*=4, 0.81%)
		Personal content (*n*=1, 0.20%)
		Outside sponsor (*n*=1, 0.20%)

From the 257 streamers who included content related to substance use, the authors inductively coded 312 reasons stated by users (e.g., multiple reasons could occur within the same Twitch livestream or description) for including this content or engaging in substance use behavior that could be mapped to 3 general themes associated with substance use. These general themes included behavioral (i.e., personal use with no evident intention of Monetary Gain [e.g., entertainment, event driven, intoxication, casual use, and content creative stream]) (94.55%, *n*=295), monetary gain (i.e., substance use content that solicited monetary contributions [e.g., bits, subs, followers, and donation]) (3.53%, *n*=11), and product promotion (i.e., direct or indirect promotion of substance use, where the product is endorsed by a brand or exchanged for nonmonetary benefits [e.g., direct substance marketing, indirect substance marketing, personal content, and outside sponsor]) (1.92%, *n*=6). In addition, the authors identified and coded 495 specific subthemes of self-reported reasons that offered deeper insights into why streamers may include substance use content in their content description or livestreams. These reasons were grouped into 13 subthemes inductively derived from the 3 general themes (Table 1, Table 3 and Table 1, Table 3 provide examples).

**Table 3:** Deidentified Examples Substance Use Detected in the Twitch Pokémon TCG Category

Substance use category	Description	Examples
Alcohol	General theme: Behavioral Subtheme: Content creative stream	
Alcohol	General theme: Product promotion Subtheme: Outside sponsor	
Tobacco/nicotine	General theme: Behavioral Subtheme: casual use	
Cannabis	General theme: Behavioral Subtheme: Event driven	
Cannabis	General Theme: Monetary gain Subtheme: Subs	

Table shows screenshots of examples of substance use on Twitch during a live Pokémon TCG broadcast.

TCG, Trading Card Game.

Profile information was reviewed for the presence of references or direct advertisement of substance use content among the 257 Pokémon TCG Twitch streamers analyzed. In total, 5.06% (*n*=13) included a reference in their profile stating their support for substance use behavior (e.g., “420/710 friendly,” meaning that they support cannabis day and oil day [generally referring to cannabis concentrates] or that the streamer self-reports using a substance during a stream) or direct promotion of a cannabis product (e.g., a marijuana-related accessory). Appendix Figures 1 and 2 (available online) provide deidentified screenshots and examples of user profiles.

The authors also evaluated the accessibility of content from the Pokémon TCG streamers identified for their fictitious account registered to an individual under the age of 18 years as well as for users not logged into a Twitch account. A total of 72.76% (*n*=187) of the Pokémon TCG streamers that included substance use content in their livestreams had no age restrictions in place for all videos and streams associated with their accounts and did not have any form of warning or disclaimer regarding the content, regardless of whether the account was registered to someone underage or not logged in.

Specific breakdown of Twitch substance use content that had no form of warnings or disclaimers by type of substance included alcohol (*n*=168), cannabis (n=3), tobacco (*n*=2), and polysubstance use (*n*=14). Access to streams that warned users on the video whose content could contain depictions of substance use were 14.79% (*n*=38) of all those reviewed, which also compares with 9.34% (*n*=24) streams where users were simply required to click a disclaimer agreeing that they would be exposed to content that could contain substance use. In total, only 2.72% (*n*=7) of streamers blocked authors’ underage account or users without a logged-in account from accessing their stream or content after they subscribed to the streamers’ content. Finally, 1 streamer’s account was banned during the data analysis process for reasons unknown to the researchers (Table 4 provides additional information).

**Table 4:** Twitch Streamers Identified for Substance Use and Access to Streams

Substance use type	Account banned^a^ (*n*=1)	No (subscribers only) (*n*=7)	Access granted (*n*=187)	Access granted (warning) (*n*=38)	Access granted (disclaimer) (*n*=24)
Alcohol	1	7	168	37	19
Cannabis	0	0	3	0	3
Nicotine/tobacco	0	0	2	0	0
Alcohol, cannabis	0	0	12	1	2
Alcohol, nicotine/tobacco	0	0	2	0	0

a Account was banned during data analysis.

## DISCUSSION

This study examined more than a thousand active and influential Pokémon TCG streamers on Twitch and found that approximately 1 in 4 (25.07%, *n*=257) depicted or promoted the use of alcohol, cannabis, tobacco, or polysubstance use. The most frequently mentioned substance discussed, depicted, promoted, or used on these streams was alcohol (90.27%), followed by a smaller number of mentions of cannabis, tobacco, and polysubstance use. The most observed general theme of these streams was behavioral-related content (e.g., primarily depicting personal substance use not for monetary gain), whereas a much smaller volume was identified that included substance use content to solicit monetary contributions and direct product promotion. In total, there were 495 mentions of streamer-specific reasons for substance use content made during these collective broadcasts, which, on the basis of data available from aggregation sites, comprise Twitch influencers who have a collective total of 6,991,926 followers with a stream time in the Pokémon TCG of 1,181,614 hours.

In addition, close to three quarters (72.76%, *n*=187) of streamers’ accounts did not prohibit underage access to account content or a livestream. This raises concerns about content classification and the effectiveness of community guidelines on Twitch. Specifically, Twitch’s content classification policy encourages but does not require users to report the use of substance use and only moderates streams on the basis of whether the mature-rated category is selected by streamers.^32^ Furthermore, community guidelines on substance use while streaming outline that certain substance use is permitted on a stream so long as the streamer is of legal age to consume (alcohol and tobacco); however, cannabis use remains a quasilegal gray area depending on the streamer’s location, although streamers nevertheless are required to self-label their broadcast under a specific drug-use label; however, only *n*=6 (28.57%) of the streamers that the authors reviewed did so.^33^

These lax community guidelines point to an overall lack of platform accountability and active content moderation of streamers who may openly violate Twitch’s substance use guidelines. This raises concerns regarding not only viewer exposure to substance use promotive content but also streamers encouraging substance use for monetary reasons (i.e., monetary donation in exchange for substance use on stream, such as requesting donations for becoming intoxicated). This is despite Twitch community’s guidelines that prohibit monetary gain for substance use. Hence, the increasing popularity of platforms such as Twitch for livestreams around various topics popular among youth and adolescents (e.g., TCGs such as Pokémon,^6^ video gaming, electronic sports, and so on) raises concerns regarding the promotion of substance use topics as observed in this study, which can easily be accessed on a smartphone, tablet, or computer by millions of users, often with no adequate age restrictions.^27^

Prior studies on Twitch have identified similar concerns, including a significant increase in brand exposure to products such as E-cigarettes, unhealthy foods, and energy drinks.^27^ The promotion cues for these products were often identified in stream titles, streamer profiles, and product placements similar to what was observed in this study. For instance, one study specifically focused on the promotion of E-cigarettes during video game streams, finding that such content contributed to the normalization of vaping and potentially encouraged uptake among youth viewers.^26^ Another Twitch study found that product placement cues, often for unhealthy items, were present for up to 20 minutes per hour of content and notably lacked clear advertising disclosures.^29^ However, this study is the first study to identify co-occurring content that includes both the promotion of Pokémon TCG (e.g., through pack battles, card reveals, giveaways, and others) and substance use behavior (e.g., drunk streams, drinking games, cannabis and smoking use, promoting substance use products).

### Limitations

Limitations of this study include a lack of generalizability of substance use across all Twitch broadcasting categories, including gaming channels, music channels, IRL (i.e., streamers broadcast their daily life or discuss it, in real life), and creative channels (i.e., culinary arts or creative activities). Strengths of this study include its use of data aggregation sites to identify influential Twitch streamers and access their archived content for content analysis purposes, along with age verification assessment. Future studies should monitor all Twitch broadcasting categories for substance use–related promotion and behavior, with a particular focus on content that is appealing and widely viewed by children and youth.

## CONCLUSIONS

Pokémon is a globally popular media franchise with a large following of children and adolescents who buy and participate in its TCGs. Twitch is a livestreaming platform that is also very popular among youth and adolescents and includes streamers who promote Pokémon TCG through card drops, rips, giveaways, and games, with roughly one fourth of these popular streamers also promoting substance use–related content with little adequate age restrictions in place. The risk of children and adolescents’ exposure to drinking games, intoxication, inebriation, smoking, and explicit product promotion while searching for and viewing Twitch Pokémon content is a digital public health challenge that needs to be further studied and addressed through appropriate policy making and platform content moderation.

## Appendix Supplementary materials

Image, application 1

## Footnotes

Supplementary material associated with this article can be found in the online version at doi:10.1016/j.focus.2025.100413.

## References

[1] Pocket Monster Red - Green. Pocket Monster Official Site. https://www.pokemon.co.jp/game/other/gb-rg/. Accessed September 12, 2024.

[2] Game Freak Inc. primary developer and co-owners of the Pokémon. https://www.gamefreak.co.jp/works/pokemon/. Accessed September 12, 2024.

[3] CNN. Pokemon mania sweeps United States. *CNN.* October 14, 1999.https://web.archive.org/web/20020205225320/http://www.cnn.com/TECH/computing/9910/14/t_t/pokemon.tt/index.html. Accessed September 12, 2024.

[4] History. The Pokémon Company. https://corporate.pokemon.co.jp/en/aboutus/history/. Accessed September 12, 2024.

[5] Conte N. (April 27, 2024). The World’s top media franchises by all-time revenue. https://www.visualcapitalist.com/the-worlds-top-media-franchises-by-all-time-revenue/.

[6] Play! Pokémon parents guide. *Pokemon.com*. https://www.pokemon.com/us/play-pokemon/about/parents-guide. Accessed September 12, 2024.

[7] Guinness World Records (2022). https://www.guinnessworldrecords.com/news/2022/4/logan-paul-owns-5-275-million-pokemon-card-after-record-breaking-trade-697189.

[8] Pokémon in figures. The Pokémon Company. https://corporate.pokemon.co.jp/en/aboutus/figures/. Accessed September 12, 2024.

[9] The Associated Press. Man who used Covid funds on a $57,000 Pokémon card gets 3 years in prison. *NBC News.* March 8, 2022.https://www.nbcnews.com/news/us-news/man-used-covid-funds-57000-pokemon-card-gets-3-years-prison-rcna19127. Accessed September 12, 2024.

[10] Serjeant J. (February 24, 2021). Gotta catch’em all: pandemic sends prices soaring for Pokemon cards. https://www.reuters.com/article/lifestyle/gotta-catch-em-all-pandemic-sends-prices-soaring-for-pokemon-cards-idUSKBN2AN1F2/.

[11] (August 23, 2021). Pokémon other collectibles hit big amid the COVID pandemic. https://www.latimes.com/business/story/2021-08-23/covid-era-pokemon-collectibles-demand-surge-causes-problems.

[12] Bogage J. (May 13, 2021). Target halts Pokémon and baseball card sales after parking lot dispute. https://www.washingtonpost.com/business/2021/05/13/target-pokemon-baseball-cards/.

[13] Mutter CJ. (February 27, 2021). Pokemon trading card Twitch category sees 3000% rise. https://gamerant.com/pokemon-trading-card-twitch-rise-popularity/.

[14] Chapman M. (January 10, 2024). Amazon’s Twitch cuts over 500 jobs attempting to turn expensive platform profitable. https://apnews.com/article/twitch-amazon-streaming-a90a279c233fa379a946b0b2e42033e6.

[15] Audience | Twitch ads. https://twitchadvertising.tv/audience/. Accessed September 12, 2024.

[16] Twitch ads - How to start advertising with Twitch | Amazon ads. https://advertising.amazon.com/library/guides/twitch-ads. Accessed September 12, 2024.

[17] Twitch usage and growth statistics: how many people use Twitch? BankLinko. https://backlinko.com/twitch-users. Accessed September 12, 2024.

[18] Chen J., Wang Y. (2021). Social media use for health purposes: systematic review. J Med Internet Res.

[19] Capriotti P., Zeler I. (2023). Analysing effective social media communication in higher education institutions. Humanit Soc Sci Commun.

[20] Özkent Y. (2022). Social media usage to share information in communication journals: an analysis of social media activity and article citations. PLoS One.

[21] Liu J., Charmaraman L., Bickham D. (2024). Association between social media use and substance use among middle and high school-aged youth. Subst Use Misuse.

[22] Rutherford B.N., Lim C.C.W., Johnson B. (2023). #TurntTrending: a systematic review of substance use portrayals on social media platforms. Addiction.

[23] Rutherford B.N., Sun T., Johnson B. (2022). Getting high for likes: exploring cannabis-related content on TikTok. Drug Alcohol Rev.

[24] Vassey J., Valente T., Barker J. (2023). E-cigarette brands and social media influencers on Instagram: a social network analysis. Tob Control.

[25] Ridout B., Campbell A., Ellis L. (2012). Off your Face(book)”: alcohol in online social identity construction and its relation to problem drinking in university students. Drug Alcohol Rev.

[26] Vassey J., Allem J.P., Barker J. (2023). E-cigarette use and promotion by social media influencers during videogame play on Twitch. Tob Control.

[27] Pollack C.C., Kim J., Emond J.A., Brand J., Gilbert-Diamond D., Masterson TD. (2020). Prevalence and strategies of energy drink, soda, processed snack, candy and restaurant product marketing on the online streaming platform Twitch. Public Health Nutr.

[28] Monitoring of marketing of unhealthy products to children and adolescents. WHO. https://www.who.int/europe/tools-and-toolkits/monitoring-of-marketing-of-unhealthy-products-to-children-and-adolescents—protocols-and-templates. Accessed September 18, 2024.

[29] Evans R., Christiansen P., Masterson T., Barlow G., Boyland E. (2024). Food and non-alcoholic beverage marketing via Fortnite streamers on Twitch: a content analysis. Appetite.

[30] Twitch Stats and Analytics - Games & Channels - SullyGnome. https://sullygnome.com/. Accessed September 12, 2024.

[31] Games and Global Statistics · TwitchTracker. https://twitchtracker.com/. Accessed September 12, 2024.

[32] Content classification labels. Twitch Help Portal. https://help.twitch.tv/s/article/content-classification-labels?language=en_US. Updated XXX. Accessed September 12, 2024.

[33] Content classification guidelines. Twitch Safety Center. https://safety.twitch.tv/s/article/Content-Classification-Guidelines?language=en_US. Accessed September 12, 2024.

