# Supplementary material for: Blasting Off Again: An Observational Study on Substance Use Content Exposure in Pokémon Twitch Streams
Source: AJPM Focus. 2025 Aug 21;4(6):100413. doi: 10.1016/j.focus.2025.100413 (PMC12547307; doi:10.1016/j.focus.2025.100413)
Supplement: Supplementary file 1 [file mmc1.docx]

**Supplemental Online Content**

This supplemental material has been provided by the authors to give readers additional information about their work.

eAppendix.

**Substance use Keywords:**

**APPENDIX:**

“420”, “710”, “🍷”, “🍻”, “🥃”, “Alcohol”, “Beer”, “Birthday”, B-day”, “Blaze it”, “Cannabis”, “Cinco de mayo”, “Drink”, “Drinko”, “Drunk”, “Hangover”, “Juice”, “Kush”, “Marijuana”, “New Years”, “Nicotine”, “Saint Patrick”, “Shots”, “Smoke”, “St. Patrick”, “Tipsy”, “Tobacco”, “Vape”, “Weed”, “White claw”, “Wine”.

**Figure 1:** Pokémon Substance Use Marketing in a Twitch Streamer’s Profile


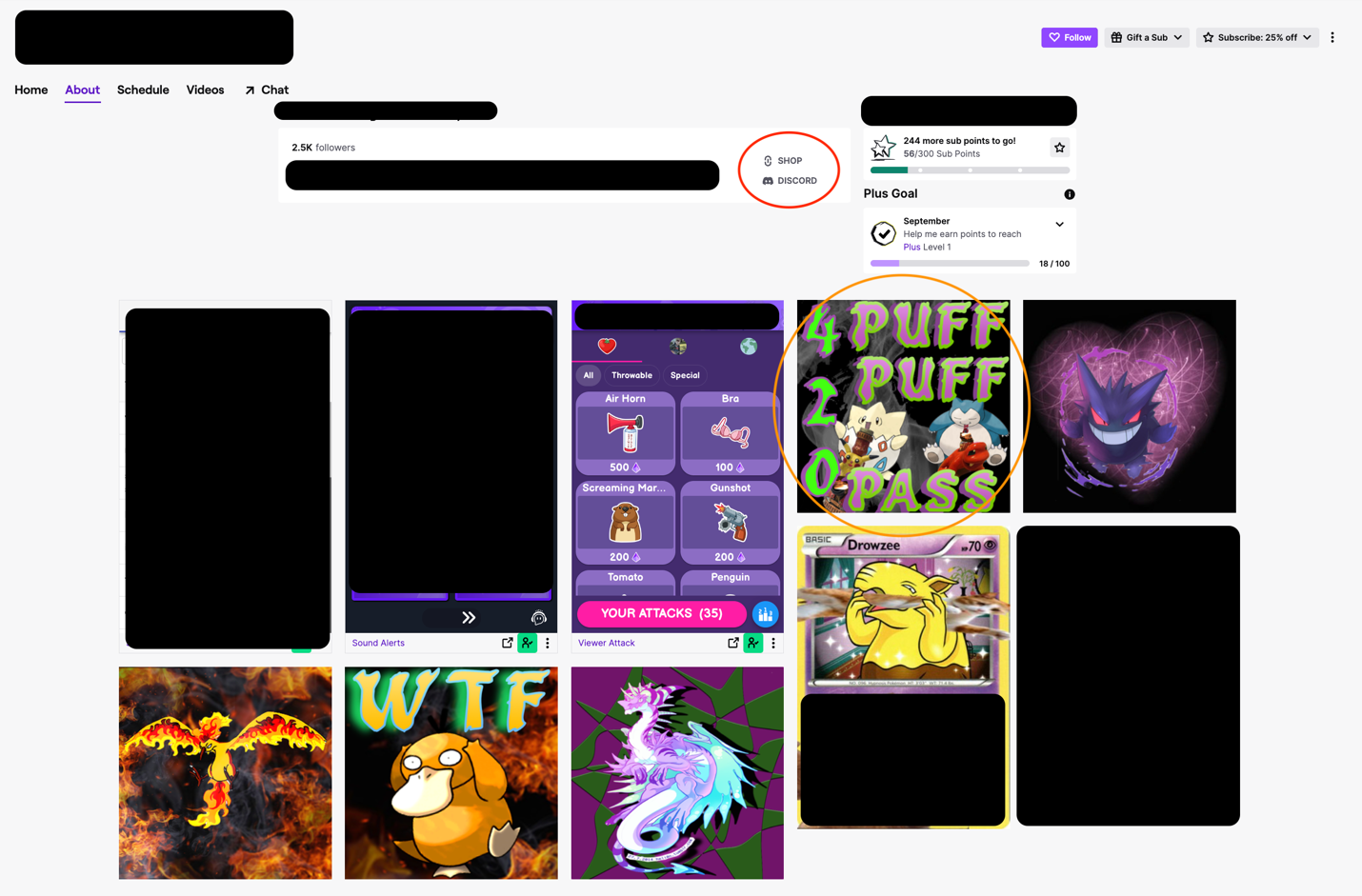


**Figure Caption:** The Figure shows an example of the marketing techniques used by Twitch Streamers in the about section. In the orange circle is an example of product placement in the streamers profile with two Pokémon (Left is Togepi; Right is Snorlax) shown vaping cannabis; in the red circle is an example of the shop in which content related to Pokémon TCG can be bought through. Throughout the profile image you can see references to Pokémon.

**Figure 2: Pokémon Imagery and Cannabis Product Marketing in a Twitch Streamer’s About Section**


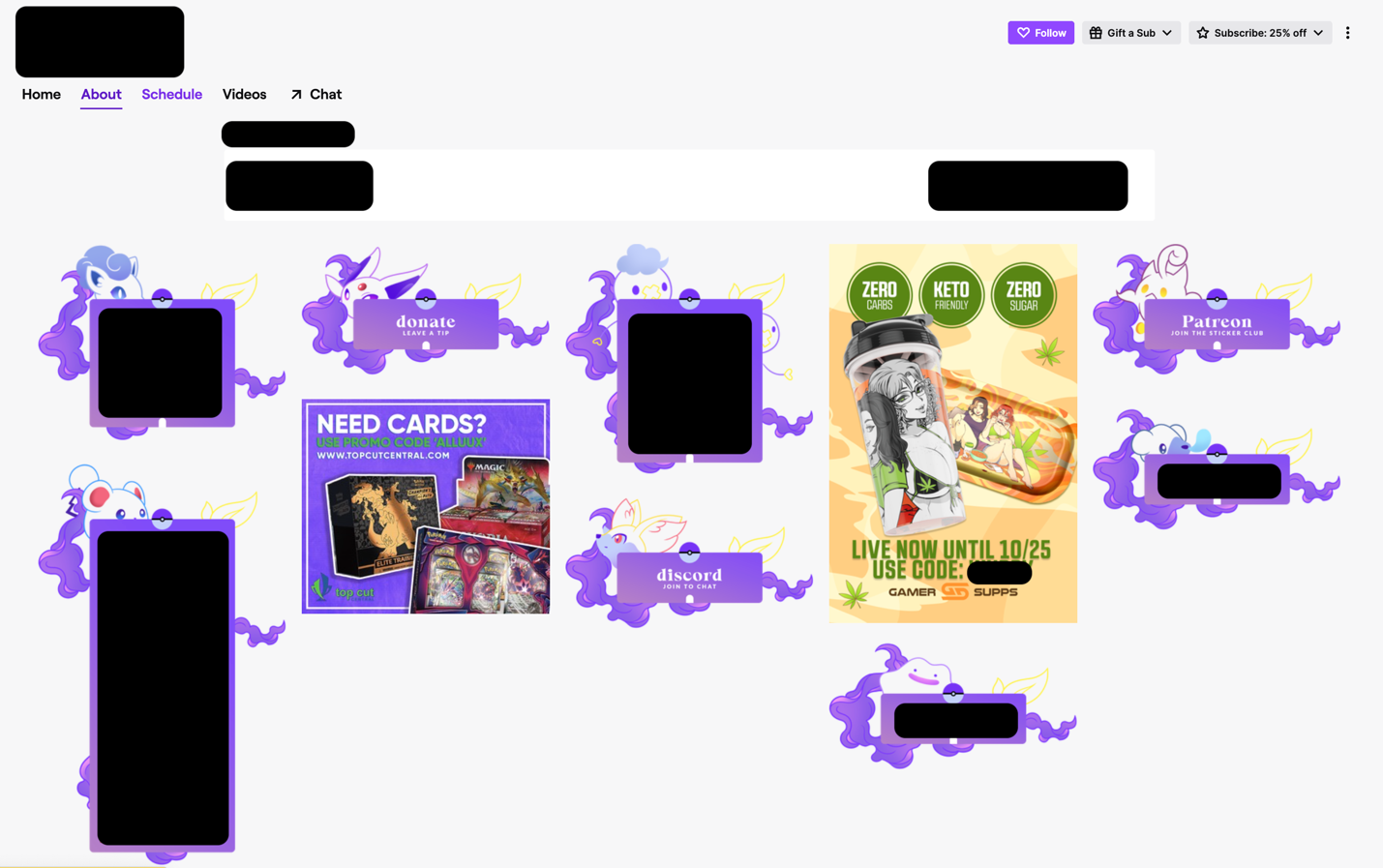


**Figure Caption:** The Figure shows an example of the marketing techniques used by Twitch Streamers in the about section. Throughout the about section, you can see depiction of different Pokémon that are colored in purple; with the purple box advertising the Pokémon TCG. In the yellow box is an example of a static brand logo image featured in the streamers about section. Clicking this image will take you to the streamers shop where a user can buy cannabis accessories (i.e., cannabis tray).
